# Lineage Switching in Acute Leukemias: A Consequence of Stem Cell Plasticity?

**DOI:** 10.1155/2012/406796

**Published:** 2012-07-19

**Authors:** Elisa Dorantes-Acosta, Rosana Pelayo

**Affiliations:** ^1^Leukemia Clinic, Mexican Children's Hospital Federico Gómez, 06720 Mexico City, DF, Mexico; ^2^Oncology Research Unit, Oncology Hospital, Mexican Institute of Social Security, 06720 Mexico City, DF, Mexico; ^3^Medical Sciences Program, National Autonomous University of Mexico, 04510 Mexico City, DF, Mexico

## Abstract

Acute leukemias are the most common cancer in childhood and characterized by the uncontrolled production of hematopoietic precursor cells of the lymphoid or myeloid series within the bone marrow. Even when a relatively high efficiency of therapeutic agents has increased the overall survival rates in the last years, factors such as cell lineage switching and the rise of mixed lineages at relapses often change the prognosis of the illness. During lineage switching, conversions from lymphoblastic leukemia to myeloid leukemia, or vice versa, are recorded. The central mechanisms involved in these phenomena remain undefined, but recent studies suggest that lineage commitment of plastic hematopoietic progenitors may be multidirectional and reversible upon specific signals provided by both intrinsic and environmental cues. In this paper, we focus on the current knowledge about cell heterogeneity and the lineage switch resulting from leukemic cells plasticity. A number of hypothetical mechanisms that may inspire changes in cell fate decisions are highlighted. Understanding the plasticity of leukemia initiating cells might be fundamental to unravel the pathogenesis of lineage switch in acute leukemias and will illuminate the importance of a flexible hematopoietic development.

## 1. Early Cell Fate Decisions in the Hematopoietic System: Unidirectional and Irreversible?

 Mature cells within the hierarchical hematopoietic system, are conventionally classified into two major lineages: lymphoid and myeloid. The lymphoid lineage consists of B, T, and natural killer (NK) cells, whereas the myeloid lineage includes erythrocytes, megakaryocytes, mast cells, granulocytes, monocytes, and macrophages. A number of subtypes of dendritic cells (DC) are generated via the pathways of lymphoid or myeloid differentiation [1–3]. Starting in the very primitive multipotential hematopoietic stem cells (HSC), lineage commitment proceeds after a gradual process of cell differentiation and concomitant series of ordered lineage exclusions. As progenitor cells progress through the pathway, their differentiation capabilities narrow, and at the point where potential limits the fate, the precursors become now-committed [4]. It is believed that once a cell is committed to a given lineage, its fate must be set due to precise combinations of lineage transcription factors and epigenetic modifications to the chromatin [5]. However, considering that hematopoiesis implies a continuing dialogue between developing cells and the surrounding microenvironmental cues [4], the unidirectional and irreversible nature of the process has been questioned by a number of findings showing redirection of cell fates through various manipulations, highlighting the plasticity of early progenitor cells [5].

**Figure 1:**
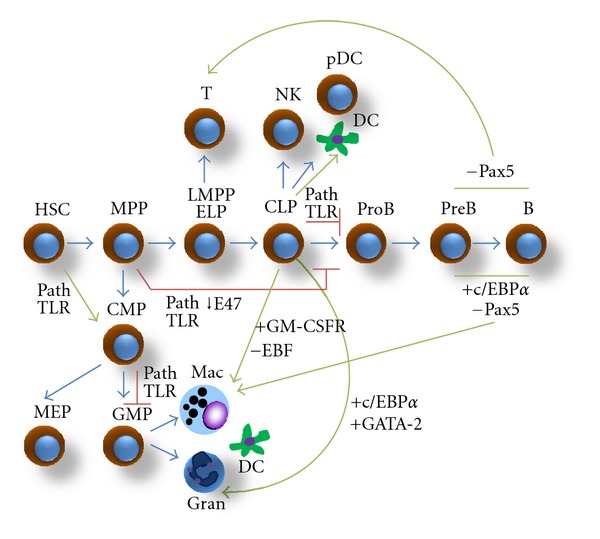
Plasticity in the hematopoietic model. Hematopoietic system is organized as a hierarchy of cell types that gradually lose multiple alternate potentials while committing to lineage fates. Ectopic expression or loss of master transcription factors in committed or developing cells, as well as the cell response to microenvironmental cues such as growth factors and pathogen products, can change fate decisions and promote cell conversions. Blue arrows follow the normal hematopoietic model, whereas green arrows follow prospective pathways of plasticity. Red lines indicate differentiation blocking by effect of pathogens or TLR ligation. HSC: hematopoietic stem cells; MPP: multipotent progenitors; LMPP: lymphoid-primed multipotent progenitors; ELP: early lymphoid progenitors; CLP, common lymphoid progenitors; TLR: Toll-like receptors; MEP: megakaryocyte-erythroid progenitors; GMP: granulocyte-monocyte progenitors; Mac: macrophage; Gran: granulocytes; DC: dendritic cells; T, T cells; NK: natural killer cells; pDC: plasmacytoid dendritic cells; GM-CSFR: granulocyte-macrophage colony-stimulating factor receptor.

 HSC give rise to multipotent progenitors (MPP) that no longer retain self-renewal and long-term reconstitution properties (Figure 1). In mice, the lymphoid differentiation program begins in the lymphoid-primed multipotent progenitors (LMPP), a population containing RAG1^+^ early lymphoid progenitors (ELP) capable of producing all lymphoid-lineage cells as well as components of the innate immune system, including plasmacytoid dendritic cells (pDC) and interferon-producing killer dendritic cells (IKDC) [3, 6, 7]. A further step on the differentiation process results in the production of common lymphoid progenitors (CLP) that are recognized as the major B and NK cell producer (Figure 1). On the other hand, MPP in turn give rise to common myeloid progenitors (CMP) that are responsible of generating granulocyte-monocyte progenitors (GMP) and megakaryocyte-erythroid progenitors (MEP) [8]. Both CLP and CMP lineage precursors have substantially lost the possibility of differentiating into the rest of the lineages and finish their developmental process producing fully committed mature cells that eventually will be exported to peripheral circulation (Figure 1). Human hematopoiesis seems to be generally consistent with the process in mice, except for the cell phenotypes. Development of myeloid and lymphoid cells from HSC also involves a stepwise progression of stem and progenitor cells in the bone marrow [9, 10]. CMP are differentiated from the fraction of multipotent progenitor cells, whereas the earliest lymphoid progenitors may be directly derived from HSC and has been recently designated as multilymphoid progenitor (MLP). A description that fully matches the definition of mouse ELP is still missing, but a counterpart of CLP efficiently differentiates into B and NK cells [10, 11]. 

 Throughout the pathways, a network of transcription factors (TF) is highly important in defining cellular fates. RUNX1, SCL, Ikaros, and GFI-1, among other TF, play a role in early development and during the specification of common myeloid progenitor from HSCs [12]. Downstream, diversification within the CMP fraction correlates with the instructive signals from GATA-1 for the megakaryocyte-erythroid lineage, while myelomonocyte cells are controlled by elevated levels of PU.1, GFI-1, c/EBP*α*, and/or c/EBP*β* [5, 8, 10]. Along the lymphoid pathway, specific NK cell regulation is conducted by Id2 and Zfp105 TF [4]. In B-versus T-lymphoid fate choice, B-cell development is determined by PU.1, E2A, EBF, and Pax5 [13], whereas access to the T-cell fate seems to depend on silencing of Pax5 and expression of GATA-3 and Notch1. Loss of E2A and EBF1 (early B-cell factor) blocks entry into the B cell program, while loss of Pax5 (paired box 5) redirects B-cells into other lineages [14]. Moreover, the enforced expression of EBF1 and Pax5 overcome the developmental block in E2A or IL-7 deficient mice, further illustrating the transcriptional hierarchy of the B-lymphoid program. Acting together with Pax5, EBF drives the expression of B-cell genes, including BLNK, CD79A, RAG, and CD19, among others. The recent report from Singh and colleagues has strikingly established the capability of EBF of repressing lineage-inappropriate genes, upstream and independently of Pax5 [15]. Loss- and gain-of-function experiments with committed lymphoid progenitors demonstrated that EBF regulates B-lymphoid versus myeloid fates by enforcing B-related genes expression while reducing the expression of myeloid-related genes, including PU.1 and EBP.

 The genetic manipulation of some of these factors has verified their participation in the lineage decisions, documenting the possibility of cell reprogramming within the hematopoietic system (Figure 1). Conditional deletion of Pax5 in mature B cells can induce conversion to different fates, including macrophages and T cells, potentially through the dedifferentiation of noncommitted progenitors [16, 17]. The absence of EBF allows early progenitors to differentiate into myeloid-lineage cells independently of Pax5, whereas sustained expression of EBF in Pax5-deficient progenitors inhibits their myeloid and T-lineage options [15]. Interestingly, the forced expression of c/EBPs in precursors of B cells results in the activation of specific myeloid genes and a rapid reprogramming to macrophages [5], while PU.1 in fully committed pre-T cells induce formation of myeloid DC, and c/EBP*α* plus PU.1 convert them to functional macrophages [18]. Iwasaki and colleagues have confirmed the importance of the TF expression timing for a proper early lineage commitment [19]. In their model, CLP could be converted to GMP, as well as basophil and mast cell progenitors by the enforced expression of c/EBP*α* and GATA-2, respectively. The order of c/EBP*α* and GATA-2 expression was shown to be critical for CLP to differentiate into eosinophils or into basophils [19].

 In addition to transcriptional regulators, inductive environmental signals, including the ones from cytokines and growth factors, are critical for the early cell fate decisions. Of note, when transduced with the GM-CSF receptor, common lymphoid progenitors are able to generate macrophages and granulocytes in response to GM-CSF [20], although this GM-CSF-induced behavior can be redirected by the constant presence of IL-7. 

 There are other examples of plasticity where progenitor cells can be redirected by extracellular factors, like during infections. Interesting findings indicate that inflammatory cues and infectious stress stimulate stem cells to leave quiescence. Moreover, these seminal cells and developing progenitors express high levels of Toll-like receptors (the receptors concerned with recognizing viral and bacterial components in mammals) and can use them to sense pathogen products, assuming alternative fates and facilitating quick differentiation of innate precursor and effector cells [3, 21–25]. Interaction of TLR2 and TLR4 with their ligands promotes the production of myeloid cells from HSC [26]. Our observations indicate similar elevated levels of TLR9 transcripts in purified fractions of lymphoid progenitors. Furthermore, the generation of DC is strongly favored at expense of B-cell production when TLR9 is ligated on CLP by DNA-CpG motifs or during herpes simplex virus 1 (HSV-1) infection [21]. Together, these data have suggested that the vigorous plasticity of progenitor's genome allow them to be reprogrammed by external signaling cues [27]. Thus, the implications of this phenomenon during lineage adjustments in hematological diseases are crucial to be determined.

## 2. The Biology of Acute Leukemias

 At present, acute leukemias (AL) are the most common cause of childhood cancer worldwide, characterized by the uncontrolled production of hematopoietic precursor cells of the lymphoid or myeloid series within the bone marrow. Of the two types of AL, acute lymphoblastic leukemia (ALL) has the highest frequency, accounting for the 85% of the cases, while acute myeloid leukemia (AML) constitutes 15% of them [28]. Nearly 80% of ALL cases have a precursor B-cell immunophenotype and approximately 15% show a T-cell immunophenotype. 

There have been several attempts to classify acute leukemias using morphologic, immunophenotypic, and cytogenetic features and the diagnosis criteria have changed according the evolution of diagnosis tools. In 1976, the French-American-British (FAB) Cooperative Group published a morphologic classification of acute leukemias [29, 30].A revision of this classification was widely used and recognized as the standard for AL classification for over 15 years. For ALL diagnosis, the FAB system defines three categories of lymphoblasts according to cell size, nuclear chromatin, nuclear shape, nucleoli, basophilia of cytoplasm, and cytoplasmic vacuolation (Table 1), whereas for the diagnosis of AML, this system includes eight subtypes (M0 to M7), each characterized by specific morphologic and histochemical features (Table 1). The FAB classification does not correlate particularly well with the immunophenotypic and cytogenetic classification. Nevertheless, Wright-Giemsa staining and application of the FAB criteria is the first step toward the diagnosis of most patients and provides guidance for additional laboratory tests.

 On the other hand, the new World Health Organization (WHO) classification proposal defines subsets of AL based on morphologic and cytogenetic characteristics [46], incorporating new information from scientific and clinical studies and adding entities that have only recently been characterized [46] (Table 1). In order to classify them, the *European Group for the Immunological Classification of Leukemia *(EGIL) [47] has created a scoring system based on the number and specificity degree of lymphoid and myeloid markers expressed by leukemic cells. In keeping with it, biphenotypic/bilineal leukemia are defined when point values are greater than 2 for myeloid and 1 for lymphoid lineages (Table 1). The WHO describes the mixed phenotype acute leukemia (MPAL) classification based on the expression of strictly specific T-lymphoid (cytoplasmic CD3) and myeloid (myeloperoxidase (MPO)) antigens, the latter shown by either flow cytometry or cytochemistry and/or clear evidence of monocytic differentiation. Because there is no single antigen strictly specific for B cells, B-cell lineage assignment in MPAL relies on the strong expression of CD19 together with another B cell-associated marker or, in cases with weak CD19, on the expression of at least 3 B-lineage markers. In addition, the WHO recognizes 2 distinct categories: MPAL with the t(9;22)(q34;q11)/BCR-ABL1 and MPAL with t(v;11q23)/MLL rearrangement. The remaining cases are designated as MPAL not otherwise specified [48].

 Although, in recent years, studies have reported important advances in the investigation of genetic, molecular, karyotypic and phenotypic aberrations that are prevalent in these diseases, the understanding of the mechanisms that damage the early programs of hematopoietic development remains poor, due in part to the fact that the precise origin of the disease and the susceptibility of primitive leukemic cells to extrinsic factors are yet to be determined [14, 49]. Even when cancer stem cells (CSC) in myeloid leukemias have been strictly depicted as the cells responsible for tumor maintenance, the identification of a rare, primitive, and malignant cell with intrinsic stem cell properties, and the ability to recapitulate the ALL has been more complicated [50] and is still on debate. Identification of leukemic clones with unrelated DJ rearrangements and cytogenetic abnormalities on lineage negative cells in ALL strongly suggest the existence of oligoclonality and oligolineage, thus the participation of primitive cells in the onset of leukemia [51, 52]. Moreover, data showing that only cells with immature phenotypes are capable of engraftment and reconstitution of leukemia in immunodeficient mice support this notion [53]. However, recent work has remarkably shown that precursor blasts at different differentiation stages can also reestablish leukemic phenotypes *in vivo*, conferring them stem cell properties [50, 54, 55] and the ability to create abnormal bone marrow microenvironments [56]. Furthermore, the combination of genomics and xenotransplant approaches has indicated unsuspected genetic diversity within subclones of leukemia initiating cells, supporting multiclonal evolution of leukemogenesis rather than lineal succession, and outlining the importance of taking account of functional plasticity. 

## 3. Lineage Switching in the Clinic

 Analysis of the expression of surface and cytoplasmic and nuclear antigens of leukemia cells has permitted their classification in function of lineage and of maturation stage. Although in the majority of the cases, markers are expressed by which specific lineages can be identified, there are situations in which both lymphoid- and myeloid-lineage markers, or T-cell and B-cell markers, coexist [30].

 Some 20–30% of patients with leukemia suffer relapses, during which it is common to find genetic alterations in the same original cell lineage (lymphoid or myeloid). In these individuals, the response to therapies for reinduction is usually of poorer quality and shorter duration. Within this high-risk group, a “lineage switch” phenomenon is occasionally observed, which occurs when acute leukemias that meet the standard FAB (French-American-British) criteria for a lineage (lymphoid or myeloid) at the time of the initial diagnosis meet the criteria for the opposite lineage upon relapse [57, 58]. A lineage switch has been considered an uncommon type of mixed leukemia [59] with a frequency between 6–9% of the cases in relapse [58]. In ALL, the most evident prognostic factor is the time to relapse. An early relapse is associated with a higher rate of nonresponse to treatment, a shorter duration of second complete remission, and a lower event-free survival rate. Most relapses in AML occur during treatment within the first year upon diagnosis. Strikingly, neonate patients that develop lineage switching, present very early relapses and poor event-free survival, that make the prognosis for these patients from variable to bad with no optimal standard treatment for them [39]. 

A lineage switch may represent either a relapse of the original clone with heterogeneity at the morphological level or high plasticity attributes, or the emergence of a new leukemic clone [36]. In attempting to explain its etiology, various mechanisms have been postulated, among which reprogramming and/or redirection of the precursor cell fate within bone marrow is prominent, as will be further discussed. Whether lineage switching is a feature of acute leukemia that promotes instability of the hematopoietic lineage or AL genome plasticity is a consequence of the leukemic transformation, are unsolved interesting issues.

## 4. The Experience of Children's Hospitals

Lineage switching has been reported to occur more frequently in children than adults [42]. Eighteen cases of pediatric lineage switch have been recorded in the literature, and the pertained information is compiled in Table 2, which may provide new insights into the mechanisms of lineage switching. 

 Cases in Table 2 are ordered by age. Even when most reports classify lineage switching cases into pediatric and adult, there is a group of patients (five out of eighteen) with congenital acute leukemia (CAL). CAL is rare and typically manifests itself within the first 4 weeks of life [31]. Interestingly, the reported clinical outcomes for this group were poor: three of them died, one was alive at the time of publication, and one more was uncertain [31–35]. Overall, approximately 40% CAL cases involve a translocation in chromosome region 11q23, including t(4;11), t(9;11), t(11;19), and other 11q23 abnormalities [60, 61]. This information is congruent with the high frequency (80%) reported for CAL lineage switching. 

 Furthermore, half of the 18 studied patients had chromosomal aberrations involving 11q rearrangements. As known, the mixed lineage leukemia (MLL) gene participate in more than 50 fusions that might be implicated in transformation of BM cells through regulation of HOX genes. Among them, the fusion MLL-AF9 is associated more commonly with acute myeloid leukemia, whereas the translocation t(4;11)(q21;q23), which produces the fusion of the MLL and AF4 genes, has been documented in up to 80% of infant ALL cases and in near to 2% of children older than 1 year of age [62]. Very recently, a retrospective observational analysis has strikingly shown the high heterogeneity in the disease biology and prognosis of the induction failure (persistence of leukemic blasts in blood, BM or extramedullary sites after 4 to 6 wk of remission-induction therapy) in ALL [63, 64]. Within the induction-failure study group, MLL/11q23 rearrangement was shown to be a poor-risk feature that was overrepresented in those patients with a highly adverse clinical outcome, recording only 16 ± 5% 10-year survival rate [64].

Despite this information, the role of genetic and chromosomal aberrations in the trigger of lineage switching is unknown, and the possibility of the first leukemic transformation occurring *in utero* during fetal hematopoiesis and the second—concomitant with lineage switch—taking place during the natural evolution of the disease are tempting.

 As described in Table 2, in some cases the original karyotype had been replaced by an entirely different abnormal karyotype, while in other patients, the lineage switch may represent a relapse of the same leukemic clone.

 Interestingly, the case of a mixed leukemia may correspond to two types of leukemia, and the phenotype switched from one lineage to another between the time of diagnosis and relapse [40]. This phenomenon could have occurred due to a clonal selection because chemotherapy eradicates the dominant clone present at diagnosis, thus permitting the expansion of a secondary clone with a different phenotype.

 Of note, most cases involve the conversion of ALL to AML, and cases of conversion from AML to ALL are extremely rare, with only five cases being reported in the English literature (Table 2). Among them, two correspond to CAL, and three correspond to pediatric AML. The time from diagnosis to conversion was approximately 1 year, and almost all patients within this group achieved remission after conversion. For our reported case of AML to ALL conversion [39], the immunocytochemistry for PAX5 suggested no expression of a transcription factor of lymphoid origin, at least at the time of remission. Moreover, between the first and second leukemias there was no evidence of lymphoid malignancy for a period of time until the patient relapsed. The absence of a lymphoid transcription factor at the beginning of surveillance suggested that the lineage switch occurred upon relapse, opening an intriguing possibility of development of *de novo *lymphoid leukemia after myeloid leukemia.

 In the switch case presented by Podgornik and colleagues, the first course of chemotherapy successfully eradicated the t(12;21). However, a second cell line with AML1 amplification may have remained latent during the time of complete remission, and then reappeared showing a different immunophenotype [43]. 

On the other hand, lineage switching may be part of the biological spectrum of mixed-lineage leukemias. Pui and colleagues have previously suggested that loss of CD10 might be related to the malignant transformation of multipotent stem cells occurring after eradication of the original stem cell line with chemotherapy. The precise significance of this finding remains unknown [65].

## 5. Potential Mechanisms of Lineage Conversion

 Several hypotheses have been suggested to explain lineage conversion in acute leukemia, but its precise mechanism remains unclear. An examination of some known physiological plasticity mechanisms may help to understand the cell and molecular biology behind this phenomenon. 

 Physiological plasticity has been defined as the capacity of changing cell fate without altering genotype [66]. Thus, epigenetic modifications might be of great importance in regulating phenotype cell conversions in response to changes in the microenvironment. 

 Accordingly, the fate of cells having the plasticity attribute as a part of their normal developmental program, is then potentially able to be redirected [66]. Under pathological circumstances, including acute leukemias, different routes might exist, other than transformation, to allow “plastic” differentiating cells to give rise to other cells different from themselves. According to Rothenberg's view, changes in cell potentials can be explained by mechanisms operating at different levels: at the cell-intrinsic level, clearly defined by transcription factors and possible epigenetic cues; and at the cell/environment interface where modification of TF activities take place in response to inductive environmental signals [4]. 

### 5.1. Bi- and Oligopotential Progenitors

 According to the classical model of hierarchical hematopoiesis, blood cells arising from HSC can be subdivided into two major lineages, a myeloerythroid and a lymphoid lineage. However, a number of recent studies indicate that the divergence lymphoid-myeloid is less abrupt than previously believed. An alternative “myeloid-based model” has been proposed by Kawamoto and Katsura in which myeloid potential is retained in erythroid, T-, and B-cell branches even after these lineages have segregated from each other [67, 68]. 

**Figure 2:**
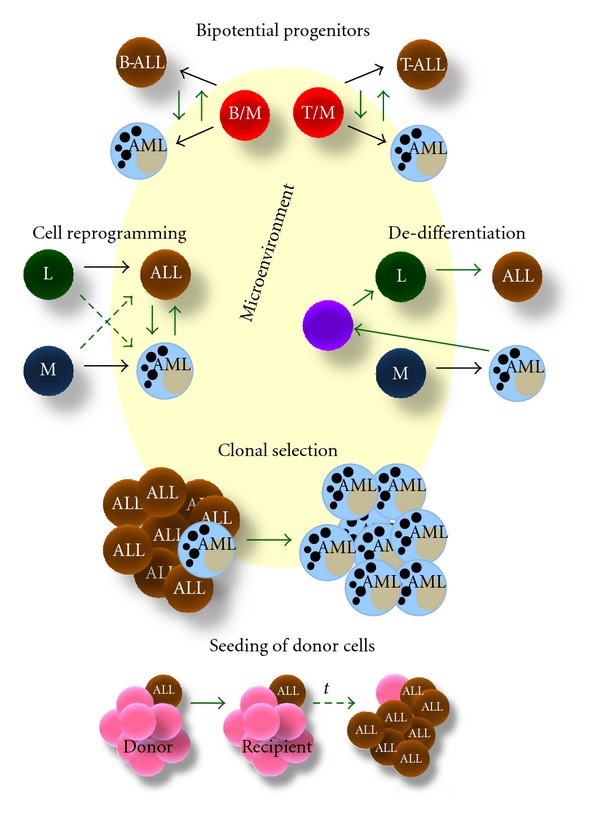
Potential mechanisms of lineage switching in acute leukemias. The existence of bipotential progenitors, cell reprogramming, dedifferentiation, clonal selection, and seeding of donor cells are proposed to participate in leukemic cell fates conversion. Microenvironment may influence all proposed mechanisms by modulating the genome plasticity of the cells and change the leukemia outcome at relapse. Black arrows follow normal differentiation, whereas green arrows indicate potential mechanisms of lineage switching. Bipotential progenitors might be responsible for fate interconversions from mixed lymphoid-myeloid leukemias. Genetic and epigenetic changes in transcription factors of fully committed or developing cells are the basis of cellular reprogramming. During dedifferentiation, a cellular change occurs in a differentiated state which in turn get back to a more primitive and less committed stage. Clonal selection is based on the existence of an oligoclonal disease, and the selection of a distinct and chemoresistant clone. In seeding of donor cell leukemia after allografts from bone marrow, a first “hit” may take place in donor followed by a second “hit” in the recipient, along with a clonal selection upon time. B/M: bipotent B and myeloid progenitor; T/M: bipotent T and myeloid progenitor; AML: acute myeloid leukemia; B-ALL: acute lymphoblastic leukemia from B precursors; T-ALL: acute lymphoblastic leukemia from T precursors; L: lymphoid progenitors; M: myeloid progenitors; *t: *time.

 The presence of early bipotential B-macrophage progenitors in the bone marrow and the fact that MLL-positive B-ALL show gene expression profiles consistent with early hematopoietic progenitors have raised the possibility that early bipotential or oligopotential progenitor cells are target for leukemogenic translocations, and constitute the origin of lineage switching events [65] (Figure 2, upper panel). Alternatively, in a subset of cases, the MLL translocation might lead to a stem/progenitor cell phenotype, irrespective of the cell lineage targeted by the translocation, and the cellular environment might allow for lineage interconversions [58]. 

 For Palomero and colleagues, leukemic transformation may occur in early progenitors and be influenced by external and internal cues [69]. Although apparently Notch signaling is essential to open the T-cell differentiation pathway but does not initiate the T-cell program itself [70], mutations occurring in the Notch1 TF in leukemic stem cells that precede both myeloid and T-lineage commitment seems to be responsible for T-cell/myeloid lineage switching, highlighting the participation of a putative common progenitor [69]. 

 Interestingly, leukemic blasts from a group of ALL and AML patients often express cell markers of more than one lineage while retaining characteristics that demonstrate a strong commitment to a single lineage, a phenomenon denominated lineage infidelity. According to St Jude Children's Hospital, AL with aberrant antigen expression can be classified into ALL that express myeloid-associated antigens (My+ALL) and AML that express lymphoid-associated antigens (Ly+AML). Large studies of patients with My+ALL and Ly+AML suggest that lineage infidelity does not have an apparent prognostic significance [71]. By contrast, mixed-lineage acute leukemias (or acute leukemias of ambiguous lineage) represent a heterogeneous category of rare, poorly differentiated acute leukemias possessing characteristics of both lymphoid and myeloid precursor cells [72]. These divergent morphologic and immunophenotypic features may be uniformly present in one blast population (biphenotypic leukemia) or may be seen on distinct blast populations in a single patient (bilineal leukemia). Leukemias that switch their lineage of origin during therapy or show poorly differentiated or undifferentiated features are also included in this category. As mentioned before, the *European Group for the Immunological Classification of Leukemia *(EGIL) [47] has created a scoring system based on the number and specificity degree of lymphoid and myeloid markers expressed by the leukemic cells. In keeping with it, a biphenotypic/bilineal leukemia is considered when point values are greater than 2 for the myeloid and then 1 for the lymphoid lineages. 

 Because the leukemic cells can aberrantly express other lineage markers, an accurate subclassification of the disease, along with a clear cut diagnosis are critical to define lineage switch. Moreover, investigation of a precursor-product relationship between bipotential progenitors and the “faithless” cells, or between bipotential progenitors and bilineal leukemias, is required and will be valuable to further understand lineage switch origins. 

### 5.2. Cell Reprogramming and Dedifferentiation

 Genetic and epigenetic activities are suggested to be directly implicated in lineage redirection, as modifications affecting chromatin structure are important for the expression of genes involved in cell fate decisions and in the maintenance of cell-differentiated states [73]. Apparently, any reprogramming implying a change towards a new cellular identity may involve epigenetic regulation [66]. 

 Using a very interesting model for instability in leukemic cells, Messina and colleagues have found an aberrant expression of activation-induced cytidine deaminase (AICDA) in BCR/ABL1+ B-ALL [74] that upregulate DNA repair/replication and cell cycle genes, and suggested its participation in the genetic instability of BCR/ABL1+ B-ALL. Lineage conversion in ALL can be promoted by significant copy number alterations of “stemness” modulators, such as deletions in peak regions from MYC, TCF3, RB1, CDKN1A, and deletions in CDKN1B [75].

 As discussed in earlier sections of this paper, lineage commitment in blood cells is controlled by transcription factors such as PU.1 and C/EBP*α* for the commitment of myeloid cells, and Notch1, GATA3, and Pax5, which mediate T- and B-cell development, respectively [5]. The ectopic expression or deletion of these master regulators mostly result in lineage reprogramming, with or without reversion of cells back to a multipotent stage [66] (Figure 2). The now-functional TF in the reprogrammed cells may be able to establish a new epigenetic program and to remove the original one.

 The introduction of c/EBP*α* into B- or T-cells converts them into functional macrophages [5, 18, 76]. While the expression of GATA-1 can reprogram common B- and T-progenitor cells to differentiate into megakaryocytic/erythroid cells [19]. Furthermore, loss of Pax5 in fully committed B cells allows them to revert to a multipotential cell and to take alternate differentiation routes upon specific stimuli [66]. An integral activity of Pax5 is pivotal for normal and neoplastic B lymphopoiesis [77, 78]. It will be crucial to investigate a correlation between genetic/epigenetic abnormalities in Pax5 and lineage switching in acute leukemias.

 In addition to genetic changes, dynamic epigenetic remodeling may take place over the course of the reprogramming processes. We have learned from *in vitro* reprogramming of somatic cells into embryonic stem cells (ESC) [79] that the ectopic expression of the four pluripotency-associated transcription factors (c-Myc, Oct-4, Klf4 and Sox2) is made possible by a variety of epigenetic changes that take place during the process, that permit the reactivation of key pluripotency-related genes, establish the appropriate bivalent chromatin domains and hypomethylate genomic heterochromatic regions. Thus, an epigenetic reorganization is central to get a cell reprogrammed [72, 80, 81].

 Of note, dedifferentiation may co-function as a mechanism for lineage conversion, where cells lacking a master TF revert to a primitive stage before committing to a second lineage fate [17]. It remains to be addressed if the cases like the *in vivo* conversion of T-ALL reported by Mantadakis et al. [44], with an early thymocyte to AML result from dedifferentiation programs.

### 5.3. Clonal Selection

 This mechanism, which would involve heterogeneous populations of developing cells, is believed to occur at relapse in patients with a persistent TEL/AML1^+^ preleukemic/leukemic clone [82]. Interestingly, karyotype analyses do not often show cytogenetic alterations, and lineage switch may represent the emergence of a new leukemic clone. Chemotherapy might suppress or eradicate the leukemic clone that is apparent at the time of diagnosis, thereby permitting the expansion of a subclone with a different phenotype (Figure 2).

### 5.4. Seeding of Donor Cells

 Although no biological cell conversion could be explained by this mechanism, its impact on the clinical lineage switch is a fact. There have been reported around 40 cases making a lineage change at relapse after hematopoietic stem cell transplantation (HSCT), as a consequence of leukemia relapse occurring in donor cells. This so-called donor cell leukemia (DCL) seems to be an uncommon and possibly underreported complication after allogeneic HSCT [83]. A major problem in the analysis of DCL is the demonstration of the donor cell origin of leukemic relapse after allogeneic transplantation, which includes cytogenetic detection of marker chromosomes, fluorescent *in situ* hybridization for the identification of sex-related chromosomes (XY-FISH), detection of Y chromosome-specific sequences (YCS-PCR) and detection of polymorphic markers like minisatellites or variable number tandem repeats (VNTRs: repeats of 10–100 bp) [84, 85]. 

Possible causes of DCL include oncogenic alteration or premature aging of transplanted donor cells in immunosuppressed individuals, aberrant homeostasis promoting transformation, impaired immune surveillance, chemotherapy-induced mutagenesis/transformation, replicative stress and a first “hit” in donor followed by second “hit” in recipient [86]. Both intrinsic cell factors and external signals from the recipient, as a proinflammatory or immunocompromised microenvironment may contribute to the leukemic clone expansion (Figure 2). 

### 5.5. The Role of the Hematopoietic Environment

 Hematopoietic stem and progenitor cells do not grow as self-supporting units; rather they are completely surrounded by the microenvironment of the BM and have a continuing dialogue with signals provided by it [4]. A network of mesenchymal cells, osteoblasts, fibroblasts, adipocytes, macrophages, endothelial cells, and reticular cells building the endosteal, vascular and reticular niches, forms a highly organized three-dimensional scaffold and supports hematopoietic differentiation [62]. Clearly, the very early fate decisions in hematopoiesis are influenced by environmental cues in physiological conditions. While it has long been recognized that intrinsic abnormalities may cause leukemia, it has also become clear that changes in microenvironment composition might lead to disease. A number of seminal studies have highlighted the microenvironment-hematopoietic relationship in leukemia, and led to propose at least three mechanisms to explain possible niche contributions to oncogenesis: competition of tumor cells for the niche, manipulation of the environment, and disruption of the HSC-niche communication [62]. How any of these alterations would allow or promote lineage switching in leukemia is currently a topical question.

 Heuser and colleagues have shown that although genetic disruption of Flt3 and c-Kit does not affect the MN1-induced leukemogenesis in the MN1 model of acute myeloid leukemia, it is important to preserve a switch from the myeloid to erythroid phenotype [87], highlighting the relevance of microenvironmental signals controlling myeloid-erythroid lineage choices. 

 Interesting studies on acute leukemias harboring MLL (mixed lineage leukemia) rearrangements have suggested that the fusion partner may instruct lineage decisions. For example, MLL-AF9 and MLL-AF6 are related more commonly with acute myeloid leukemia (AML), while the fusion MLL-AF4 and MLL-ENL has been mostly documented in ALL [88]. The capability of MLL-GAS7 cells to generate distinct leukemias in mice models, including an acute biphenotypic leukemia, supports the existence of a multipotent leukemia-initiating cell that may give rise to both AML and ALL [89]. Moreover, using a human-based MLL leukemia mouse model, the role of microenvironment has been shown to be critical to the lineage outcome, with manipulation of the *in vivo* cytokine milieu influencing the commitment of both lineage-restricted and multipotent LIC [90]. Again, these findings underline the plasticity of leukemic MLL-target cells and their critical vulnerability to environmental cues.

 Finally, our prior observations suggest that in normal conditions, human and mouse HSC and lymphoid progenitors in bone marrow respond to stimulation by microbial components through Toll-like receptors (TLR), thereby redirecting their differentiation potentials [3, 21] (Vadillo et al., unpublished data). Thus, there is a strong possibility that their TLR-expressing counterparts in leukemia represent the beginning of instability of the lineage. The *in vitro* TLR ligation on CD34+ cells from ALL pediatric patients induce cell proliferation and redirection of cell fates (Dorantes-Acosta et al., unpublished data). Along with recurrent infections, increasing evidence suggests the prevalence of inflammatory environments in hematological abnormalities such as acute leukemias [56, 91], remaining to be addressed if overproduction of inflammatory cytokines impacts the HSC niches and can stimulate aberrant cell fate decisions.

### 5.6. Prospective Signaling Pathways in Lineage Conversion

 A comprehensive model for the molecular and signaling pathways involved in both nonleukemic and leukemic cell fate conversions is not yet available. Canonical routes participating in the regulation of lineage decisions may function as platforms for abnormal activities of transcription factors, oncoproteins or rearranged genes. MLL trithorax domain participate in the methylation of H3K4, activating the transcription of leukemogenesis- and cell fate-associated genes like HOX [13]. HOX deregulation is the most relevant factor for MLL fusion-induced leukemogenesis. HOX proteins, in particular HOXA9 and its partner MEIS1, are oncoproteins substantially overexpressed in leukemias, can function through activation of the protooncogene c-Myb [92]. On the other hand, an elegant model of MLL-AF9-induced AML showing the significance of the microenvironment in providing instructive signals for leukemic lineage fates, has suggested that the signaling through the small GTPase Rac1 pathway is critical to leukemia development within this particular lineage promiscuity scenario [86].

Proliferation and apoptosis are defining features of the hematopoietic development, and the NF-*κ*B signaling pathway participates in their regulation [93]. The effects of NEMO inactivation in both mice and human strengthen the role of NF-*κ*B in lymphopoiesis—in the absence of NEMO-dependent NF*κ*B signaling, B and T cells fail to develop. However, whether NF-*κ*B contributes to early lineage cell decisions or just play a survival role is yet to be determined [93].

EBF1 is critical to B-lineage commitment, driving the expression of genes relevant to B-cell differentiation and function at both genetic and epigenetic levels. EBF alterations are common in patients with poor outcomes and are particularly frequent (25%) in relapsed children [14]. A recent report from Sigvardsson has shown an increase lineage plasticity and low expression of Ebf-1 on committed lymphoid progenitors in the absence of IL-7, supporting the notion that Ebf is crucial for lineage restriction [94]. Despite their findings position this transcription factor downstream of IL-7 in the developmental hierarchy, the role of STAT5 in fate conversions is uncertain. A regulatory circuit with EBF as determinant of B-lymphoid versus myeloid fates has been proposed from the Ebf^−/−^ reporter mouse model, where EBF regulates expression of myeloid-related transcription factors and can reprogram early progenitor cells [15]. EBF induction is controlled by PU.1, E2A, and IL-7R, and its promoter is responsive to STAT5, which is conventionally phosphorilated as result of JAK activation. Interestingly, genetic alterations of members of the JAK family are particularly prominent in acute leukemias [95]. Of note, STAT5 is also a critical node in the signaling pathway of BCR/ABL, and we have recently learned from the model of BCR/ABL-tumour initiation that its activity may influence the ultimate leukaemia phenotype [96].

## 6. Concluding Remarks

Lineage switching is an example of the lineage heterogeneity that exists in acute leukemias, representing a relapse of the original clone with high attributes of plasticity, or the emergence of new leukemic clones. As this phenomenon clearly correlates with very bad prognosis and resistance to therapy, further sequential phenotypic and cytogenetic studies may yield valuable insights into the mechanisms of leukemic recurrence and possible implications for treatment selection. Despite tremendous progress in the knowledge of the pathogenesis of acute leukemias, much remains to be addressed about the mechanisms driving lineage switching at relapse. Aberrant function of specific fusion genes and surrounding microenvironmental cues might guide leukemia phenotype conversion through modulation of plasticity within leukemia initiating cells. Moreover, clinical features could play important roles in establishing environmental scenarios proper for cell conversion events. Although we have much to learn about what controls and coordinate the mechanisms of action in lineage exclusions and switching, clearly leukemia-initiating cells are considerably more plastic in their developmental potential than previously envisioned, challenging the notion of limited lineage fates in these diseases.

## Figures and Tables

**Table 1:** Criteria defining acute leukemias according to current classifications.

	Acute lymphoblastic leukemia	Acute myeloid leukemia	Acute leukemias of ambiguous lineage and biphenotypic leukemia
WHO 2008	B lymphoblastic leukemia/lymphoma	Acute myeloid leukemia and related neoplasms	Acute leukemias of ambiguous lineage
	(i) B lymphoblastic leukemia/lymphoma, NOS	(i) Acute myeloid leukemia with recurrent genetic abnormalities	(i) Acute undifferentiated leukemia
	(ii) B lymphoblastic leukemia/lymphoma with recurrent genetic abnormalities	AML with t(8;21)(q22;q22); RUNX1-RUNX1T1	(ii) Mixed phenotype acute leukemia with t(9;22)(q34;q11.2); BCR-ABL1
	B lymphoblastic leukemia/lymphoma with (9;22)(q34;q11.2); BCR-ABL 1	AML with inv(16)(p13.1q22) or t(16;16)(p13.1;q22); CBFB-MYH11	(iii) Mixed phenotype acute leukemia with t(v;11q23); MLL rearranged
	B lymphoblastic leukemia/lymphoma with t(v;11q23); MLL rearranged	APL with t(15;17)(q22;q12); PML-RARA	(iv) Mixed phenotype acute leukemia, B-myeloid, NOS
	B lymphoblastic leukemia/lymphoma with t(12;21)(p13;q22) TEL-AML1 (ETV6-RUNX1)	AML with t(9;11)(p22;q23); MLLT3-MLL	(v) Mixed phenotype acute leukemia, T-myeloid, NOS
	B lymphoblastic leukemia/lymphoma with hyperdiploidy	AML with t(6;9)(p23;q34); DEK-NUP214	(vi) Provisional entity: natural killer (NK) cell lymphoblastic leukemia/lymphoma
	B lymphoblastic leukemia/lymphoma with hypodiploidy	AML with inv(3)(q21q26.2) or t(3;3)(q21;q26.2); RPN1-EVI1	
	B lymphoblastic leukemia/lymphoma with t(5;14)(q31;q32) IL3-IGH	AML (megakaryoblastic) with t(1;22)(p13;q13); RBM15-MKL1	
	B lymphoblastic leukemia/lymphoma with t(1;19)(q23;p13.3); TCF3-PBX1	Provisional entity: AML with mutated NPM1	
	T lymphoblastic leukemia/lymphoma	Provisional entity: AML with mutated CEBPA	
		(ii) Acute myeloid leukemia with myelodysplasia-related changes	
		(iii) Therapy-related myeloid neoplasms	
		(iv) Acute myeloid leukemia, not otherwise specified	
		AML with minimal differentiation	
		AML without maturation	
		AML with maturation	
		Acute myelomonocytic leukemia	
		Acute monoblastic/monocytic leukemia	
		Acute erythroid leukemia	
		Pure erythroid leukemia	
		Erythroleukemia, erythroid/myeloid	
		Acute megakaryoblastic leukemia	
		Acute basophilic leukemia	
		Acute panmyelosis with myelofibrosis	
		(v) Myeloid sarcoma	
		(vi) Myeloid proliferations related to Down syndrome	
		Transient abnormal myelopoiesis	
		Myeloid leukemia associated with Down Syndrome	
		(vii) Blastic plasmacytoid dendritic cell Neoplasm	

FAB	L1: lymphoblasts are usually smaller, with scanty cytoplasm and inconspicuous nucleoli	M0: undifferentiated. Undifferentiated, large, agranular blasts; >90% blasts MPO−; SBB−	
	L2: lymphoblasts are larger, and they demonstrate considerable heterogeneity in size, prominent nucleoli and more abundant cytoplasm	M1: acute myeloblastic, no maturation. Undifferentiated; >90% blasts; <10% promyelocytes/monocytes. MPO+;SSB+; PAS−	
	L3: lymphoblasts notable for their deep cytoplasmic basophilia, large, frequently display prominent cytoplasmic vacuolation, morphologically identical to Burkitt's lymphoma cells	M2: acute myeloblastic with maturation. ≥30% and ≤89% blasts; >105 promyelocytes, myelocytes; <20% monocytic cells. MPO+; SSB+; PAS−	
		M3: acute promyelocytic-hypergranular type. >20% abnormal hypergranular promyelocytes; Auer rods common. MPO+; SSB+; PAS−	
		M3v: acute promyelocytic-hypogranular variant. Fine granularity of cytoplasm in promyelocytes; folded nuclei. MPO+; SSB+; PAS−	
		M4: acute myelomonocytic. ≥30% blasts on nonerythroid series; >20% but <80% monocytic cells; blood monocytes >5 × 10^9^/ L; lysozyme >3 × normal. MPO+; NASDA+	
		M4eo: acute myelomonocytic with eosinophilia. >5% abnormal eosinophils with basophilic granules. MPO+; NASDA+ eosinophils; PAS+	
		M5a: acute monocytic. >80% of monocytic cells = monoblasts; rest are promonocytes/monocytes. MPO+; NASDA+	
		M5b: acute monocytic with differentiation. <80% of monocytic cells are monoblasts; rest are promocytes/monocytes. MPO+; NASDA+	
		M6: acute erythroleukemia. >30% of nonerythroid cells are blasts; >50% of marrow are erythroblasts. PAS+; ringed sideroblasts with iron stain	
		M7: acute megakaryoblastic. >30% of nonerythroid cells are megakaryoblasts; cytoplasmic blebs; myelofibrosis common. MPO−; SBB−; NASDA+ platelet MPO+ by EM	

EGIL			Scoring system for the definition of acute biphenotypic leukemias
			Scoring lineages points
			2: B-lymphoid (CD79a, CD22, cyIgM) T-lymphoid (CD3) myeloid, (MPO)
			1: B-lymphoid (CD10, CD19), T-lymphoid (CD2, CD5), myeloid (CD13, CD33)
			0.5: B-lymphoid (TdT), T-lymphoid (TdT, CD7), myeloid (CD14, CD15, CD11b, CD11c)

NOS: not otherwise specified, MPO: myeloperoxidase, SSB: Sudan Black B, PAS: periodic acid-Schiff, EM: electron microscopy, NASDA: naphthol-ASD chloroacetate, TdT: Terminal deoxynucleotide transferase, and cy: cytoplasmic.

**Table 2:** Pediatric cases of lineage switching in acute leukemias.

Number of case	Age	Sex	Diagnosis			Important findings			Time from first diagnosis to relapse	Time from second diagnosis to relapse	Criteria for lineage switching	Clinical outcome	References
			1st diagnosis	2nd diagnosis at relapse	3rd diagnosis at second relapse	1st diagnosis	2nd diagnosis at relapse	3rd diagnosis at second relapse					
1	Neonate	F	ALL L1	AML M5	—	46 XX t(1;6), t(4;11) CD19+ CD22+ CD79a+, MPO− CD34− CD117−	46 XX t(1;6), t(4;11) CD19− CD22− CD79a− CD14+ CD33+ CD13+	—	Day 100 after induction of chemotherapy	—	Morphologic and immunophenotypic	Died	[31]

2	At birth	F	AML M5	B-ALL L1	—	t(9;11)	Karyotype NR	—	12 mo	—	Morphologic	Alive	[32]

3	At birth	F	AML M5	B-ALL	—	t(4:11) MPO− CD33+ CD13+ HLA-DR+ CD14+	t(4:11) CD34+ CD19+ CD22+ HLADR+ CD10−	—	18 dy	—	Morphologic and immunophenotypic	Died	[33]

4	12 dy	?	B-ALL	AML M5	—	MLL rearrangement	MLL rearrangement	—	7 dy	—	Morphologic	NR	[34]

5	21 dy	M	Pro-B ALL	AML	—	t(4;11) MPOX− CD10– CD19+ CD22+ CD34+ CD38+	t(4;11) CD20+ CD13+ CD14+ CD15+ CD33+ CD41+ CD61+	—	8 dy	—	Morphologic and immunophenotypic	Died	[35]

6	3 mo	F	Pre-B cell ALL L1	AML M4	—	t(4;11) PAS+ MPO− ANBE− CD19+ CD34+ TdT+ CD33+	FISH with MLL signal MPO+ ANBE+ CD2+ CD13+ CD14+ CD33+ CD41+ CD65+	—	2 mo	—	Morphologic and immunophenotypic	Alive after allo-HSCT	[36]

7	9 mo	M	ALL	AML M5b	—	t(11;16)	t(11;16)	—	8 mo	—	Morphologic	Died	[37]

8	15 mo	M	pro-B ALL L1	AML M0	—	46 XY. CD19+ HLA-DR+	46 XY t(9;11) MPO− CD33+ cyCD13+ cyCD33+ CD117+	—	76 mo	—	Morphologic, immunophenotypic and cytogenetic	Alive after allo-HSCT	[36]

9	25 mo	?	AML	ALL L1	—	46 XY (11q23) PAS+ MPO−	Normal karyotype	—	1 yr	—	Morphologic and cytogenetic	Alive	[38]

10	4 yr	M	AML M5	ALL pro-B	—	Normal karyotype PAX 5 negative when patient was under surveillance CD10− CD19− CD13+ CD14+ CD15+	Normal karyotype CD10+ CD19+ CD13− CD14− CD15−	—	9 mo	—	Morphologic and immunophenotypic	Died	[39]

11	4 yr	F	ALL L1	AML mo	BM: ALL L1 CNS: AML mo	Normal karyotype BM: MPO– BM: CD13+ CD19+ D22+ CD33+ CD38+ CD34+ HLA DR+	BM: MPO+	Karyotype 47XX+ 18 BM: MPO– BM: CD13− CD19+ D22 NR CD33+ CD38 NR CD34 NR HLA DR+	2 yr after complete remission	1 mo	Morphologic and immunophenotypic	Died	[40]

12	6 yr	M	B-lineage common cell ALL L1	AML M4	—	56 XY HLA-DR+ TdT+ CD10+ CD19+ CD22+	46 XY t(8;16) MPO+ ANBE+ HLA-DR+ CD13+ CD14+ CD33+	—	9 mo	—	Morphologic, immunophenotypic, and cytogenetic	Alive	[36]

13	7 yr	F	B-lineage ALL L2	T cell ALL	AML M1	46, XX MPO− HLA-DR+, cyIgM+ CD33.+ PAS+ NSE−	Trisomy 13 CD2+ CD5+ CD7+ CD34+ HLA-DR+ CD33+	ANBE−. MPO+. CD13+, CD33+, CD34+ HLA-DR+ CD7+	14 mo	45 dy	Morphologic, immunophenotypic and cytogenetic	Died	[36]

14	8 yr	M	AML	ALL	—	Karyotype NR	Normal karyotype	—	13 mo	—	Morphologic	Alive	[41]

15	9 yr	M	ALL L1	AML M4	—	56 XY	46 XY t(8;16)	—	9 mo	—	Morphologic and cytogenetic	Alive	[42]

16	13 yr	F	Common B-cell ALL	AML M4/M5	—	t(12;21) amplification of RUNX1	Amplification of RUNX1	—	5 yr	—	Morphologic	Alive	[43]

17	16 yr	F	T-cell ALL	AML M0	—	46 XX MPO− CD7+ CD4− CD8− CD1− TdT+	46 ~ 62, XX, + X, MPO− CD19+ CD117+ CD33+ CD34+ CD56+	—	13 mo	—	Morphologic, immunophenotypic, and cytogenetic	Died	[44]

18	20 yr	M	T-ALL	AML	—	52 XY MPO− cytCD3+, CD5+ CD2+ TdT+ CD7+ CD3− CD1a− CD10− CD33− CD117− CD19− CD13^±^	MPO− CD117+ CD33+ CD13+ CD56+ TdT− CD7− cyCD3− CD2− CD5− CD19+	—	21 mo	—	Morphologic and immunophenotypic	Died	[45]

M: male, F: female, BM: bone marrow, CNS: central nervous system, FISH: fluorescence *in situ* hybridization, ANBE: *α*-naphthyl-butyrate esterase, PAS: periodic acid-Schiff, NSE: nonspecific esterase, MPO: myeloperoxidase, TdT: terminal deoxynucleotide transferase, yr: year, mo: months, dy: days, cy: cytoplasmic, and NR: not reported.

## Abbreviations

AICDA: Activation-induced cytidine deaminase
AL: Acute leukemias
ALL: Acute lymphoblastic leukemia
AML: Acute myeloid leukemia
ANBE: *α*-naphthyl-butyrate esterase
B-ALL: B-cell acute lymphoblastic leukemia
BM: Bone marrow
B/M: Bipotent B and myeloid progenitor
CAL: Congenital acute leukemia
CLP: Common lymphoid progenitors
CMP: Common myeloid progenitors
CNS: Central nervous system
CSC: Cancer stem cells
DC: Dendritic cells
DCL: Donor cell leukemia
EGIL: European Group for the Immunological Classification of Leukemia
ELP: Early lymphoid progenitors
ESC: Embryonic stem cells
FAB: French-American-British
FISH: Fluorescence in situ hybridization
GM-CSF: Granulocyte-macrophage colony-stimulating factor
GM-CSFR: Granulocyte-macrophage colony-stimulating factor receptor
GMP: Granulocyte-monocyte progenitors
Gran: Granulocytes
HSC: Hematopoietic stem cells
HSCT: Hematopoietic stem cell transplantation
HSV-1: Herpes simplex virus 1
IKDC: Interferon-producing killer dendritic cells
LMPP: Lymphoid-primed multipotent progenitors
Ly+AML: AML with lymphoid-associated antigens
MEP: Megakaryocyte-erythroid progenitors
MLL: Mixed lineage leukemia
MLP: Multilymphoid progenitor
MPAL: Mixed phenotype acute leukemia
MPO: Myeloperoxidase
MPP: Multipotent progenitors
My+ALL: ALL with myeloid-associated antigens
NASDA: Naphthol-ASD chloroacetate
NK: Natural killer cells
NSE: Nonspecific esterase
PAS: Periodic Acid Schiff
pDC: Plasmacytoid dendritic cells
SSB: Sudan Black B
T-ALL: T-cell acute lymphoblastic leukemia
TdT: Terminal deoxynucleotide transferase
TF: Transcription factors
TLR: Toll-like receptors
T/M: Bipotent T and myeloid progenitor
VNTRs: Variable number tandem repeats
WHO: World Health Organization
XY-FISH: Fluorescent *in situ* hybridization for sex-related chromosomes
YCS-PCR: Y chromosome-specific sequences.

## References

[1] Pelayo R, Welner R, Perry SS (2005). Lymphoid progenitors and primary routes to becoming cells of the immune system. *Current Opinion in Immunology*.

[2] Pelayo R, Miyazaki K, Huang J, Garrett KP, Osmond DG, Kincade PW (2006). Cell cycle quiescence of early lymphoid progenitors in adult bone marrow. *Stem Cells*.

[3] Welner RS, Pelayo R, Kincade PW (2008). Evolving views on the genealogy of B cells. *Nature Reviews Immunology*.

[4] Rothenberg EV (2011). T cell lineage commitment: identity and renunciation. *Journal of Immunology*.

[5] Xie H, Orkin SH (2007). Immunology: changed destiny. *Nature*.

[6] Pelayo R, Hirose J, Huang J (2005). Derivation of 2 categories of plasmacytoid dendritic cells in murine bone marrow. *Blood*.

[7] Welner RS, Pelayo R, Garrett KP (2007). Interferon-producing killer dendritic cells (IKDCs) arise via a unique differentiation pathway from primitive c-kitHiCD62L+ lymphoid progenitors. *Blood*.

[8] Iwasaki H, Akashi K (2007). Hematopoietic developmental pathways: on cellular basis. *Oncogene*.

[9] Blom B, Spits H (2006). Development of human lymphoid cells. *Annual Review of Immunology*.

[10] Doulatov S, Notta F, Laurenti E, Dick JE (2012). Hematopoiesis: a human perspective. *Cell Stem Cell*.

[11] Doulatov S, Notta F, Eppert K, Nguyen LT, Ohashi PS, Dick JE (2010). Revised map of the human progenitor hierarchy shows the origin of macrophages and dendritic cells in early lymphoid development. *Nature Immunology*.

[12] Baba Y, Pelayo R, Kincade PW (2004). Relationships between hematopoietic stem cells and lymphocyte progenitors. *Trends in Immunology*.

[13] Perez-Vera P, Reyes-Leon A, Fuentes-Panana EM (2011). Signaling proteins and transcription factors in normal and malignant early B cell development. *Bone Marrow Research*.

[14] Pelayo R, Dorantes-Acosta E, Vadillo E, Fuentes-Panana E, Pelayo R (2012). From HSC to B-lymphoid cells in normal and malignant hematopoiesis. *Advances in Hematopoietic Stem Cell Research*.

[15] Pongubala JM, Northrup DL, Lancki DW (2008). Transcription factor EBF restricts alternative lineage options and promotes B cell fate commitment independently of Pax5. *Nature Immunology*.

[16] Nutt SL, Heavey B, Rolink AG, Busslinger M (1999). Commitment to the B-lymphoid lineage depends on the transcription factor Pax5. *Nature*.

[17] Cobaleda C, Jochum W, Busslinger M (2007). Conversion of mature B cells into T cells by dedifferentiation to uncommitted progenitors. *Nature*.

[18] Laiosa CV, Stadtfeld M, Xie H, de Andres-Aguayo L, Graf T (2006). Reprogramming of committed T cell progenitors to macrophages and dendritic cells by C/EBP alpha and PU.1 transcription factors. *Immunity*.

[19] Iwasaki H, Mizuno SI, Arinobu Y (2006). The order of expression of transcription factors directs hierarchical specification of hematopoietic lineages. *Genes and Development*.

[20] Kondo M, Scherer DC, Miyamoto T (2000). Cell-fate conversion of lymphoid-committed progenitors by instructive actions of cytokines. *Nature*.

[21] Welner RS, Pelayo R, Nagai Y (2008). Lymphoid precursors are directed to produce dendritic cells as a result of TLR9 ligation during herpes infection. *Blood*.

[22] Baldridge MT, King KY, Boles NC, Weksberg DC, Goodell MA (2010). Quiescent haematopoietic stem cells are activated by IFN-*γ* in response to chronic infection. *Nature*.

[23] Boiko JR, Borghesi L (2012). Hematopoiesis sculpted by pathogens: toll-like receptors and inflammatory mediators directly activate stem cells. *Cytokine*.

[24] De Luca K, Frances-Duvert V, Asensio MJ (2009). The TLR1/2 agonist PAM3CSK4 instructs commitment of human hematopoietic stem cells to a myeloid cell fate. *Leukemia*.

[25] Sioud M, Floisand Y (2007). TLR agonists induce the differentiation of human bone marrow CD34+ progenitors into CD11c+ CD80/86+ DC capable of inducing a Th1-type response. *European Journal of Immunology*.

[26] Nagai Y, Garrett KP, Ohta S (2006). Toll-like receptors on hematopoietic progenitor cells stimulate innate immune system replenishment. *Immunity*.

[27] Heyworth PG, Noack D, Cross AR (2002). Identification of a novel NCF-1 (p47-phox) pseudogene not containing the signature GT deletion: significance for A47 degrees chronic granulomatous disease carrier detection. *Blood*.

[28] Perez-Saldivar ML, Fajardo-Gutiérrez A, Bernáldez-Ríos R (2011). Childhood acute leukemias are frequent in Mexico City: descriptive epidemiology. *British Medical Journal*.

[29] Bennett JM, Catovsky D, Daniel M-T (1976). Proposals for the classification of the acute leukaemias. French-American-British (FAB) co-operative group. *British Journal of Haematology*.

[30] Bennett JM, Catovsky D, Daniel MT (1985). Proposed revised criteria for the classification of acute myeloid leukemia. A report of the French-American-British Cooperative Group. *Annals of Internal Medicine*.

[31] Jiang JG, Roman E, Nandula SV, Murty VVS, Bhagat G, Alobeid B (2005). Congenital MLL-positive B-cell acute lymphoblastic leukemia (B-ALL) switched lineage at relapse to acute myelocytic leukemia (AML) with persistent t(4;11) and t(1;6) translocations and JH gene rearrangement. *Leukemia and Lymphoma*.

[32] Shimizu H, Culbert SJ, Cork A, Iacuone JJ (1989). A lineage switch in acute monocytic leukemia. A case report. *American Journal of Pediatric Hematology/Oncology*.

[33] Krawczuk-Rybak M, Zak J, Jaworowska B (2003). A lineage switch from AML to ALL with persistent translocation t(4;11) in congenital leukemia. *Medical and Pediatric Oncology*.

[34] Ridge SA, Cabrera ME, Ford AM (1995). Rapid intraclonal switch of lineage dominance in congenital leukaemia with a MLL gene rearrangement. *Leukemia*.

[35] Sakaki H, Kanegane H, Nomura K (2009). Early lineage switch in an infant acute lymphoblastic leukemia. *International Journal of Hematology*.

[36] Park M, Koh KN, Kim BE (2011). Lineage switch at relapse of childhood acute leukemia: a report of four cases. *Journal of Korean Medical Science*.

[37] Stasik C, Ganguly S, Cunningham MT, Hagemeister S, Persons DL (2006). Infant acute lymphoblastic leukemia with t(11;16)(q23;p13.3) and lineage switch into acute monoblastic leukemia. *Cancer Genetics and Cytogenetics*.

[38] Bernstein ML, Esseltine DW, Emond J, Vekemans M (1986). Acute lymphoblastic leukemia at relapse in a child with acute myeloblastic leukemia. *American Journal of Pediatric Hematology/Oncology*.

[39] Dorantes-Acosta E, Arreguin-Gonzalez F, Rodriguez-Osorio CA, Sadowinski S, Pelayo R, Medina-Sanson A (2009). Acute myelogenous leukemia switch lineage upon relapse to acute lymphoblastic leukemia: a case report. *Cases Journal*.

[40] Ikarashi Y, Kakihara T, Imai C, Tanaka A, Watanabe A, Uchiyama M (2004). Double leukemias simultaneously showing lymphoblastic leukemia of the bone marrow and monocytic leukemia of the central nervous system. *American Journal of Hematology*.

[41] Emami A, Ravindranath Y, Inoue S (1983). Phenotypic change of acute monocytic leukemia to acute lymphoblastic leukemia on therapy. *American Journal of Pediatric Hematology/Oncology*.

[42] Chung HJ, Park CJ, Jang S, Chi HS, Seo EJ, Seo JJ (2007). A case of lineage switch from acute lymphoblastic leukemia to acute myeloid leukemia. *The Korean Journal of Laboratory Medicine*.

[43] Podgornik H, Debeljak M, Žontar D, Černelč P, Prestor VV, Jazbec J (2007). RUNX1 amplification in lineage conversion of childhood B-cell acute lymphoblastic leukemia to acute myelogenous leukemia. *Cancer Genetics and Cytogenetics*.

[44] Mantadakis E, Danilatou V, Stiakaki E, Paterakis G, Papadhimitriou S, Kalmanti M (2007). T-cell acute lymphoblastic leukemia relapsing as acute myelogenous leukemia. *Pediatric Blood and Cancer*.

[45] van den Ancker W, Terwijn M, Regelink J (2009). Uncommon lineage switch warrants immunophenotyping even in relapsing leukemia. *Leukemia Research*.

[46] Vardiman JW, Thiele J, Arber DA (2009). The 2008 revision of the World Health Organization (WHO) classification of myeloid neoplasms and acute leukemia: rationale and important changes. *Blood*.

[47] Bene MC, Castoldi G, Knapp W (1995). Proposals for the immunological classification of acute leukemias. *Leukemia*.

[48] Matutes E, Pickl WF, Veer MV (2011). Mixed-phenotype acute leukemia: clinical and laboratory features and outcome in 100 patients defined according to the WHO 2008 classification. *Blood*.

[49] Akashi K (2009). Lymphoid lineage fate decision of hematopoietic stem cells. *Annals of the New York Academy of Sciences*.

[50] Bomken S, Fišer K, Heidenreich O, Vormoor J (2010). Understanding the cancer stem cell. *British Journal of Cancer*.

[51] Davi F, Gocke C, Smith S, Sklar J (1996). Lymphocytic progenitor cell origin and clonal evolution of human B-lineage acute lymphoblastic leukemia. *Blood*.

[52] Stankovic T, Weston V, McConville CM (2000). Clonal diversity of Ig and T-cell receptor gene rearrangements in childhood B-precursor acute lymphoblastic leukaemia. *Leukemia and Lymphoma*.

[53] Cox CV, Diamanti P, Evely RS, Kearns PR, Blair A (2009). Expression of CD133 on leukemia-initiating cells in childhood ALL. *Blood*.

[54] Heidenreich O, Vormoor J (2009). Malignant stem cells in childhood ALL: the debate continues. *Blood*.

[55] le Viseur C, Hotfilder M, Bomken S (2008). In childhood acute lymphoblastic leukemia, blasts at different stages of immunophenotypic maturation have stem cell properties. *Cancer Cell*.

[56] Colmone A, Amorim M, Pontier AL, Wang S, Jablonski E, Sipkins DA (2008). Leukemic cells create bone marrow niches that disrupt the behavior of normal hematopoietic progenitor cells. *Science*.

[57] Gagnon GA, Childs CC, LeMaistre A (1989). Molecular heterogeneity in acute leukemia lineage switch. *Blood*.

[58] Stass S, Mirro J, Melvin S (1984). Lineage switch in acute leukemia. *Blood*.

[59] Imataki O, Ohnishi H, Yamaoka G (2010). Lineage switch from precursor B cell acute lymphoblastic leukemia to acute monocytic leukemia at relapse. *International Journal of Clinical Oncology*.

[60] Bresters D, Reus ACW, Veerman AJP, Van Wering ER, Van Der Does-Van Den Berg A, Kaspers GJL (2002). Congenital leukaemia: the Dutch experience and review of the literature. *British Journal of Haematology*.

[61] Fernandez MC, Weiss B, Atwater S, Shannon K, Matthay KK (1999). Congenital leukemia: successful treatment of a newborn with t(5;11)(q31;q23). *Journal of Pediatric Hematology/Oncology*.

[62] Purizaca J, Meza I, Pelayo R (2012). Early lymphoid development and microenvironmental cues in B-cell acute lymphoblastic leukemia. *Archives of Medical Research*.

[63] Rabin KR (2012). Attacking remaining challenges in childhood leukemia. *The New England Journal of Medicine*.

[64] Schrappe M, Hunger SP, Pui C-H (2012). Outcomes after induction failure in childhood acute lymphoblastic leukemia. *The New England Journal of Medicine*.

[65] Pui CH, Raimondi SC, Behm FG (1986). Shifts in blast cell phenotype and karyotype at relapse of childhood lymphoblastic leukemia. *Blood*.

[66] Cobaleda C (2010). Reprogramming of B cells. *Methods in Molecular Biology*.

[67] Kawamoto H, Katsura Y (2009). A new paradigm for hematopoietic cell lineages: revision of the classical concept of the myeloid-lymphoid dichotomy. *Trends in Immunology*.

[68] Bell JJ, Bhandoola A (2008). The earliest thymic progenitors for T cells possess myeloid lineage potential. *Nature*.

[69] Palomero T, McKenna K, O-Neil J (2006). Activating mutations in NOTCH1 in acute myeloid leukemia and lineage switch leukemias. *Leukemia*.

[70] Weerkamp F, Luis TC, Naber BAE (2006). Identification of Notch target genes in uncommitted T-cell progenitors: no direct induction of a T-cell specific gene program. *Leukemia*.

[71] Rubnitz JE, Onciu M, Pounds S (2009). Acute mixed lineage leukemia in children: the experience of St Jude Children’s Research Hospital. *Blood*.

[72] Weir EG, Ansari-Lari MA, Batista DAS (2007). Acute bilineal leukemia: a rare disease with poor outcome. *Leukemia*.

[73] Zardo G, Cimino G, Nervi C (2008). Epigenetic plasticity of chromatin in embryonic and hematopoietic stem/ progenitor cells: therapeutic potential of cell reprogramming. *Leukemia*.

[74] Messina M, Chiaretti S, Iacobucci I (2011). AICDA expression in BCR/ABL1-positive acute lymphoblastic leukaemia is associated with a peculiar gene expression profile. *British Journal of Haematology*.

[75] Strauss R, Hamerlik P, Lieber A, Bartek J (2012). Regulation of stem cell plasticity: mechanisms and relevance to tissue biology and cancer. *Molecular Therapy*.

[76] Graf T (2002). Differentiation plasticity of hematopoietic cells. *Blood*.

[77] Falini B, Mason DY (2002). Proteins encoded by genes involved in chromosomal alterations in lymphoma and leukemia: clinical value of their detection by immunocytochemistry. *Blood*.

[78] Smith E, Sigvardsson M (2004). The roles of transcription factors in B lymphocyte commitment, development, and transformation. *Journal of Leukocyte Biology*.

[79] Wernig M, Meissner A, Foreman R (2007). In vitro reprogramming of fibroblasts into a pluripotent ES-cell-like state. *Nature*.

[80] Takahashi K, Yamanaka S (2006). Induction of pluripotent stem cells from mouse embryonic and adult fibroblast cultures by defined factors. *Cell*.

[81] Jaenisch R, Young R (2008). Stem cells, the molecular circuitry of pluripotency and nuclear reprogramming. *Cell*.

[82] Panzer-Grümayer ER, Cazzaniga G, Van Der Velden VHJ (2005). Immunogenotype changes prevail in relapses of young children with TEL-AML1-positive acute lymphoblastic leukemia and derive mainly from clonal selection. *Clinical Cancer Research*.

[83] Ruiz-Argüelles GJ, Ruiz-Argüelles A, Garcés-Eisele J (2007). Donor cell leukemia: a critical review. *Leukemia and Lymphoma*.

[84] Schichman SA, Suess P, Vertino AM, Gray PS (2002). Comparison of short tandem repeat and variable number tandemrepeat genetic markers for quantitative determination of allogeneic bone marrow transplant engraftment. *Bone Marrow Transplantation*.

[85] Buño I, Nava P, Simón A (2005). A comparison of fluorescent in situ hybridization and multiplex short tandem repeat polymerase chain reaction for quantifying chimerism after stem cell transplantation. *Haematologica*.

[86] Flynn CM, Kaufman DS (2007). Donor cell leukemia: insight into cancer stem cells and the stem cell niche. *Blood*.

[87] Heuser M, Park G, Moon Y (2010). Extrinsic signals determine myeloid-erythroid lineage switch in MN1 leukemia. *Experimental Hematology*.

[88] Muntean AG, Hess JL (2008). MLL-AF9 leukemia stem cells: hardwired or taking cues from the microenvironment?. *Cancer Cell*.

[89] So CW, Karsunky H, Passegué E, Cozzio A, Weissman IL, Cleary ML (2003). MLL-GAS7 transforms multipotent hematopoietic progenitors and induces mixed lineage leukemias in mice. *Cancer Cell*.

[90] Wei J, Wunderlich M, Fox C (2008). Microenvironment determines lineage fate in a human model of MLL-AF9 leukemia. *Cancer Cell*.

[91] Espinoza-Hernández L, Cruz-Rico J, Benítez-Aranda H (2001). In vitro characterization of the hematopoietic system in pediatric patients with acute lymphoblastic leukemia. *Leukemia Research*.

[92] Mueller D, García-Cuéllar MP, Bach C, Buhl S, Maethner E, Slany RK (2009). Misguided transcriptional elongation causes mixed lineage leukemia. *PLoS Biology*.

[93] Hayden MS, Ghosh S (2011). NF-*κ*B in immunobiology. *Cell Research*.

[94] Tsapogas P, Zandi S, Åhsberg J (2011). IL-7 mediates Ebf-1-dependent lineage restriction in early lymphoid progenitors. *Blood*.

[95] Chen E, Staudt LM, Green AR (2012). Janus kinase deregulation in leukemia and lymphoma. *Immunity*.

[96] Kovacic B, Hoelbl A, Litos G (2012). Diverging fates of cells of origin in acute and chronic leukaemia. *EMBO Molecular Medicine*.

